# LncRNA MALAT1 regulates smooth muscle cell phenotype switch via activation of autophagy

**DOI:** 10.18632/oncotarget.23230

**Published:** 2017-12-14

**Authors:** Tie-Feng Song, Li-Wen Huang, Ying Yuan, Hui-qin Wang, Hong-Peng He, Wen-Jian Ma, Li-Hong Huo, Hao Zhou, Nan Wang, Tong-Cun Zhang

**Affiliations:** ^1^ Key Laboratory of Industrial Fermentation Microbiology, Ministry of Education and Tianjin, College of Biotechnology, Tianjin University of Science and Technology, Tianjin 300457, P.R. China; ^2^ Institute of Biology and Medicine, Wuhan University of Science and Technology, Wuhan 430000, P.R. China

**Keywords:** vascular smooth muscle cells, MALAT1, phenotype switching, autophagy, miR142-3p

## Abstract

Vascular smooth muscle cells (VSMCs), switching from a differentiated to a proliferative phenotype, contribute to various vascular diseases. However, the role of long noncoding RNA metastasis-associated lung adenocarcinoma transcript 1 MALAT1 in the phenotype switching of VSMCs remains unclear. Here, we report that the knockdown of MALAT1 promotes the transformation of smooth muscle cells from a proliferative phenotype to a differentiated phenotype. MALAT1 knockdown inhibited cellular proliferation and migration, leading to significant cell cycle arrest in the G2 phase. MALAT1 was downregulated in bone morphogenetic protein-7 (BMP-7)-induced cellular differentiation, while MALAT1 was upregulated in platelet-derived growth factor-BB (PDGF-BB)-induced cellular proliferation. PDGF induced the transformation of smooth muscle cells into a proliferative phenotype accompanied by an increase in autophagy. The downregulation of MALAT1 attenuated PDGF-BB-induced proliferation and migration by inhibiting autophagy. MALAT1 could act as a competing endogenous RNA (ceRNA) to regulate autophagy-related 7 (ATG7) gene expression by sponging miR142-3p. The present study reveals a novel mechanism by which MALAT1 promotes the transformation of smooth muscle cells from contraction to synthetic phenotypes.

## INTRODUCTION

Vascular smooth muscle cells (VSMCs) are highly specialized and differentiated cells in adult animals. The principal function of VSMCs is contraction, which permits the regulation of vessel tone and diameter and thus controls blood pressure and blood flow distribution. VSMCs within the adult blood vessel proliferate at an extremely low rate and exhibit low synthetic activity. A number of SMC-selective or SMC-specific genes have been identified as markers of mature-differentiated VSMCs. These include smooth muscle isoforms of the proteins that comprise the contractile apparatus, including SM α-actin (ACTA2) [1–3], SM myosin heavy chain (MHC) [1–4], h1-calponin [5, 6], SM-22 [6, 7], and smoothelin [8]. Phenotypic switching of vascular smooth muscle cells (VSMCs) and subsequent proliferation contributed to various vascular diseases, including atherosclerosis, in-stent restenosis, transplant vasculopathy, and vein bypass graft failure [9]. The phenotypic switching of VSMCs is characterized by the decreased gene expression of contractile markers and the increased proliferation and migration of cells.

Platelet-derived growth factor (PDGF) is a potent mitogen for VSMCs and plays a major role in inducing the phenotypic switching of VSMCs from contractile to proliferative states. During early atherogenesis, the vessel wall exhibits synthetic and secretory activities for a number of cytokines and growth factors, which modulate the phenotypic characteristics of VSMCs through autocrine and paracrine mechanisms [10–12]. In particular, acute vascular injury promotes the increased synthesis and release of PDGF from several vascular cells, including endothelial cells, VSMCs, activated monocytes, and monocyte-derived macrophages [12–14]. *In vitro*, PDGF induces phenotypic switching in VSMCs from the contractile to the proliferative state. The increased expression of PDGF and PDGF receptors was also observed in human coronary arteries after angioplasty [15, 16]. Importantly, PDGF undergoes phenotypic transformation from a contraction to a proliferative status in VSMCs, reflecting the unique properties of phenotypic plasticity in VSMCs.

Bone morphogenetic protein (BMP) represents the largest group of growth factors in the transforming growth factor (TGF) superfamily [17–19]. BMPs inhibit the proliferation of serum-induced pulmonary artery smooth muscle cells (PASMCs) [20, 21]. In addition, BMP-7 stimulates the maintenance of the phenotype of aortic VSMCs [22]. In the present study, we treated VSMCs with BMP-7 and observed that BMPs effectively induced a systolic phenotype in VSMCs and upregulated contractile gene expression.

Long noncoding RNAs (lncRNAs) represent a class of RNA molecules with a length of more than 200 nt, which does not encode a protein but regulates the expression level of genes at various levels in the form of RNA. Studies have shown that lncRNA play important roles in a variety of biological processes, such as cell proliferation, differentiation, apoptosis, autophagy, immune responses, and angiogenesis [23–25]. Metastasis-associated lung adenocarcinoma transcript 1 (MALAT1), associated with the metastasis of lung tumors, promotes the proliferation, migration and invasion of several tumor cells, such as hepatocellular, ovarian cancer, andgastriccancer [26–29]. Although the effect of MALAT1 ontumorcells has been studied, the effect of MALAT1 on phenotypic switching of smooth muscle cells is unclear.

Autophagy is a highly conserved catabolic course, delivering cytoplasmic material and organelles to lysosomes for degradation. Autophagy, a multi-step and complex process, is regulated by approximately 30 autophagy-related proteins (ATGs) in conjunction with various signaling pathways [30]. Among these ATGs, autophagy-related protein 7 (ATG7) plays a crucial role in autophagosome formation and vesicle progression and is essential for the activation of autophagy [31]. On one hand, ATG7 functions as an E1-like ligase that conjugates autophagy-related protein 5 (ATG5) to autophagy-related protein 12 (ATG12), a required step for autophagosome formation [32]. On the other hand, ATG7 converts LC3-I, an immature and cytosolic protein, into LC3-II, a mature autophagosomal membrane protein [33]. ATG7-deficient mice die within 1 day from birth because of an impaired autophagy pathway [34]. Autophagy has been implicated in the modulation of VSMCs functions, including growth, migration, contraction/relaxation, and differentiation [35]. Autophagy may also regulate the phenotypic switching of VSMCs. Indeed, the treatment of smooth muscle cells with PDGF upregulates synthetic gene expression, VSMCs proliferation and autophagy induction [36]. However, the role and potential mechanism of MALAT1 in the activation of autophagy during VSMCs proliferation remains unknown.

Here, we examined the role of the long non-coding RNA MALAT1 in vascular smooth muscle cell phenotype transformation. Knocking down MALAT1 using small interfering RNA (siRNA) targeting this molecule promoted the differentiation but inhibited the cell proliferation and migration of VSMCs. Treatment of VSMCs with BMP-7 resulted in the downregulation of MALAT1 expression and consequent induction of a contractile phenotype in these cells, whereas treatment with PDGF-BB increased MALAT1 expression and promoted cell proliferation. PDGF-BB upregulated the expression of genes associated with the proliferation and migration of VSMCs, while knockdown of MALAT1 inhibited PDGF-induced cell proliferation and migration. These results initially indicated a role for MALAT1 in the phenotypic transformation of VSMCs. Furthermore, we explored the mechanism by which MALAT1 regulated the phenotypic transformation of VSMCs. The results demonstrated that PDGF induced autophagy in VSMCs, while MALAT1 knockdown inhibited autophagy in these cells. As a competitive endogenous RNA (ceRNA), MALAT1 promoted the switching of VSMC to a proliferative phenotype. Thus, MALAT1 and ATG7 may represent target genes of miR142-3p. Collectively, MALAT1 regulated the level of ATG7 and activated autophagy through competitively binding to miR142-3p, leading to the conversion of VSMCs from a contractile to a proliferative phenotype.

## RESULTS

### Knockdown of MALAT1 promoted the transformation of smooth muscle cells from proliferative phenotype to differentiated phenotype

**Figure 1:**
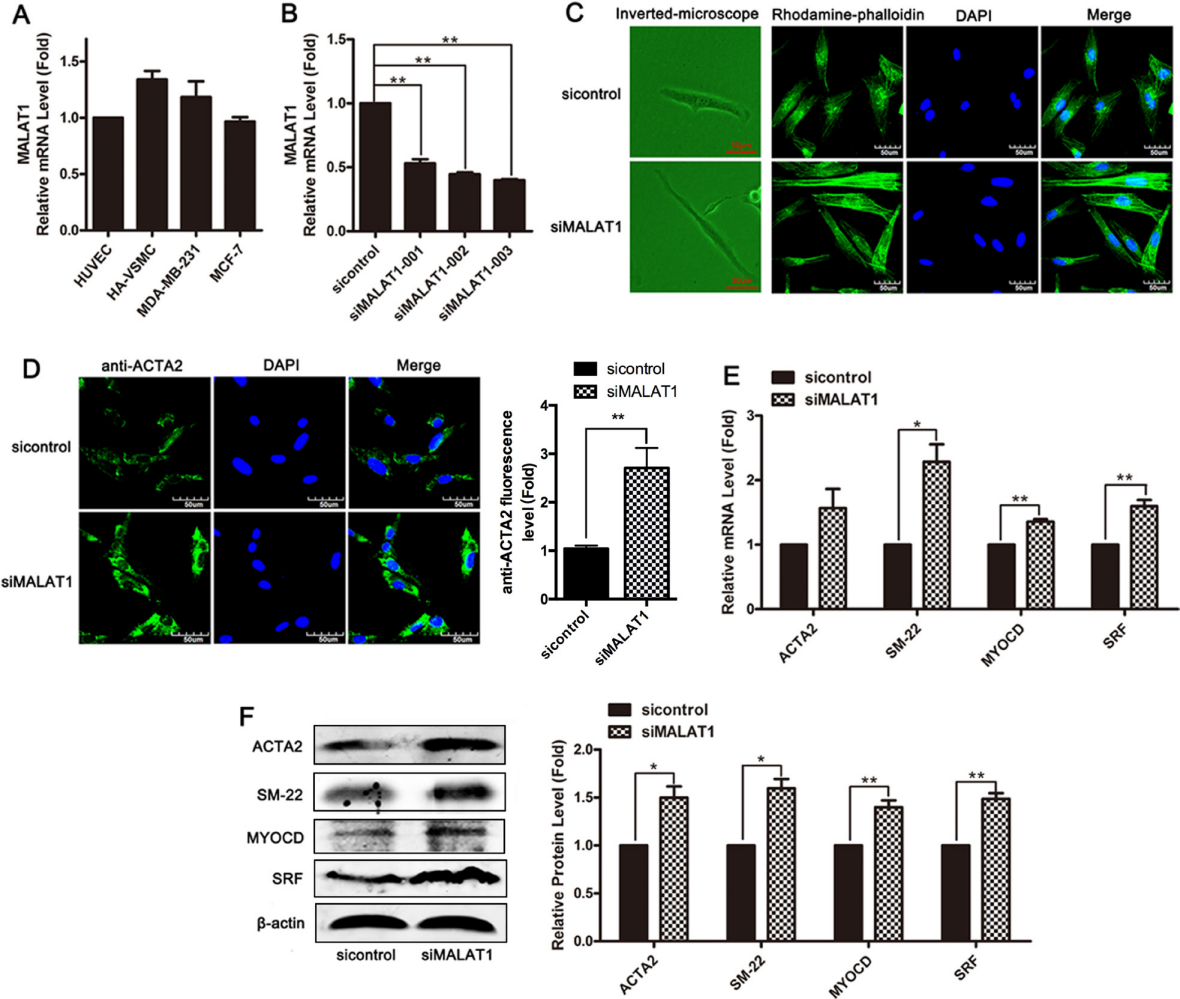
Knockdown of MALAT1 promoted the transformation of smooth muscle cells from a proliferative phenotype to a differentiated phenotype (**A**) The expression of MALAT1 in several cell lines, including HUVECs, HA-VSMCs, MDA-MB-231 and MCF-7 cells was detected using qRT-PCR. (**B**) SiRNA-mediated knockdown experiments were performed in VSMCs. Three siRNAs, which target different sequences of MALAT1 or control siRNA (sicontrol), were transfected into smooth muscle cells for 24 h, and subsequently the level of MALAT1 was detected using qRT-PCR. ^**^P < 0.01. (**C**) Left channel: morphological changes of VSMCs transfected with siMALAT1 or sicontrol were observed using phase contrast microscopy. Right channel: phalloidin staining was performed in VSMCs transfected with siMALAT1 or sicontrol. (**D**) Immunofluorescence staining showed the expression of ACTA2 (green) in MALAT1 knockdown VSMCs. The nuclei were stained with DAPI (blue). Ruler, 60 μm. (**E**) Twenty h after transfection with siMALAT1, the mRNA level of ACTA2, SM22, myocardin and SRF was detected using qRT-PCR. ^*^P < 0.05. (**F**) The expression of ACTA2, SM22, myocardin and SRF in VSMCs transfected with siMALAT1 was detected using western blotting.

First, we evaluated the level of MALAT1 in several cell lines, including HUVECs, HA-VSMCs and MDA-MB-231 and MCF-7 cells. MALAT1 was highly expressed in smooth muscle cells (Figure 1A). To investigate the role of MALAT1 in phenotype transformation of smooth muscle cell, the level of endogenic MALAT1 was knocked down in VSMCs using a small interfering RNA (siRNA)-based silencing approach, and subsequently cellular morphology was observed using phalloidin staining. Three siRNAs targeting different MALAT1 sequences or control siRNA (sicontrol) were transfected into smooth muscle cells for 24 h, and subsequently the level of MALAT1 was detected using qRT-PCR. As shown in Figure 1B, transfection with any one of three siRNAs significantly decreased the levels of MALAT1; thus, siMALAT1-003 was used in subsequent experiments. Compared with the control group, VSMCs transfected with siMALAT1 were thinned and stenosed (Figure 1C). Increased actin filaments in VSMCs transfected with siMALAT1 were observed using phalloidin staining (Figure 1C).

To assess the effect of MALAT1 on the differentiation of HVSMCs, the expression of SMC-specific contractile genes, including ACTA2, SM-22, myocardin (MYOCD) and SRF, was detected in siMALAT1-transfected HVSMCs through immunostaining, qRT-PCR and Western blotting. VSMCs transfected with siMALAT1 showed the increased expression of the contractile gene ACTA2 (Figure 1D). As shown in Figure 1E and 1F, MALAT1 knockdown resulted in the upregulation of the expression of differentiation genes, including ACTA2, SM-22, myocardin and SRF, in HVSMCs. These results showed that MALAT1 knockdown promoted the differentiation of VSMCs.

### Downregulation of MALAT1 inhibited smooth muscle cell proliferation

**Figure 2:**
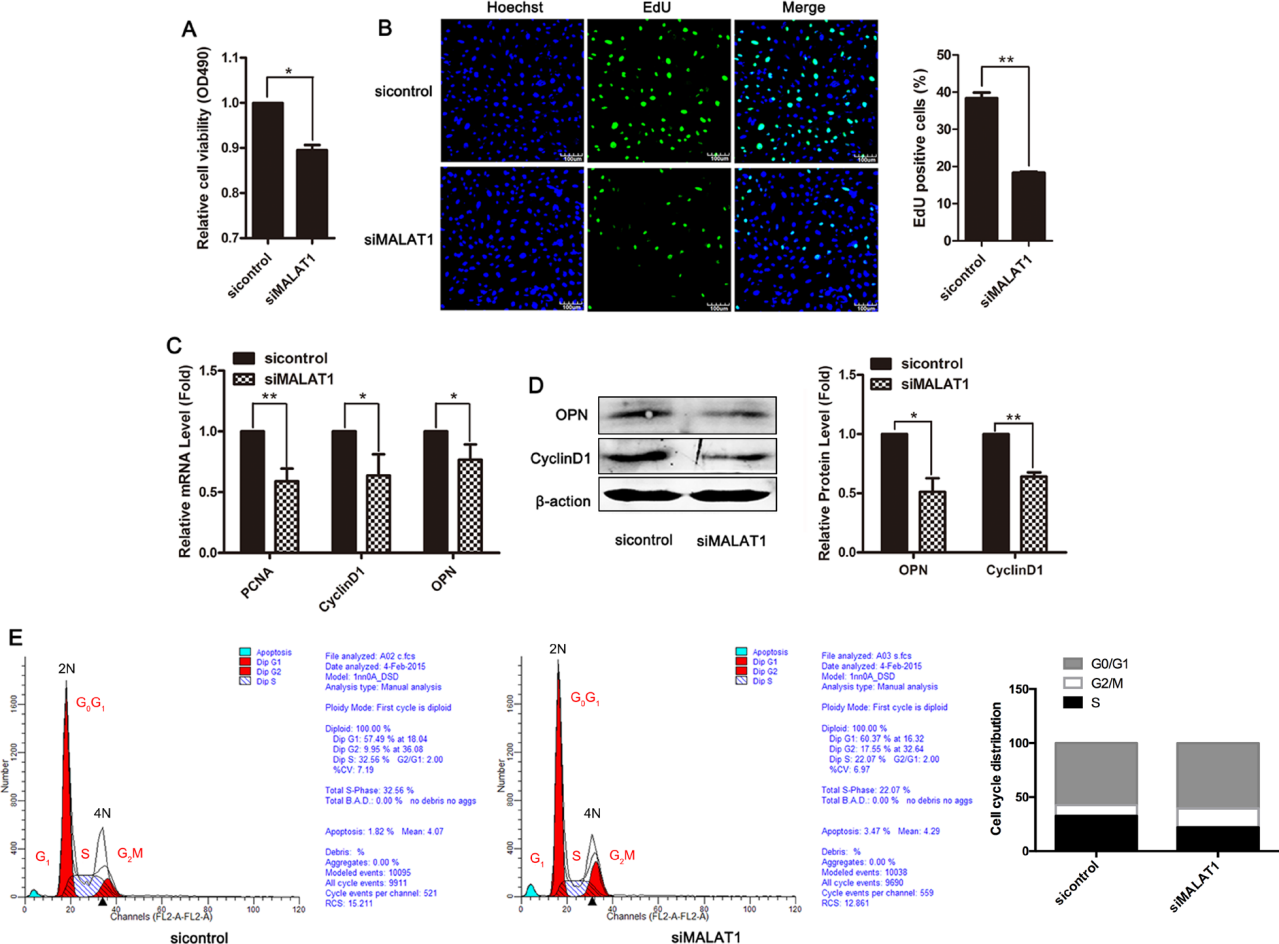
Downregulation of MALAT1 inhibited smooth muscle cell proliferation VSMCs were transfected with siMALAT1 or control siRNA for 24 h (**A**) The viability of cells was detected using MTT assay. (**B**) The proliferation ability of cells was evaluated using EdU staining. The level of proliferation markers PCNA, CyclinD1 and OPN was examined using qRT-PCR (**C**) and western blotting (**D**). ^*^P < 0.05; ^**^P < 0.01. (**E**) Cell cycle distribution was analyzed using flow cytometry at 24 h after transfection with siMALAT1.

To assess the role of MALAT1 in the proliferation of VSMCs, siRNA-mediated knockdown experiments were performed, and subsequently cell viability and proliferation were detected using MTT assays and EdU staining. As shown in Figure 2A and 2B, transfection with siMALAT1 inhibited smooth muscle cell viability and proliferation. Downregulation of MALAT1 in VSMCs reduced the expression of the proliferation markers PCNA, Cyclin D1 and the OPN gene (Figure 2C and 2D). To further confirm the effect of MALAT1 on smooth muscle cell proliferation, cell cycle distribution was analyzed using flow cytometry at 24 h after transfection with siMALAT1. After MALAT1 knockdown in VSMCs, significant cell cycle arrest in the G2 phase was observed (Figure 2E). These results indicated that the downregulation of MALAT1 inhibited smooth muscle cell proliferation.

### MALAT1 knockdown attenuated the migration of *VSMCs*

**Figure 3:**
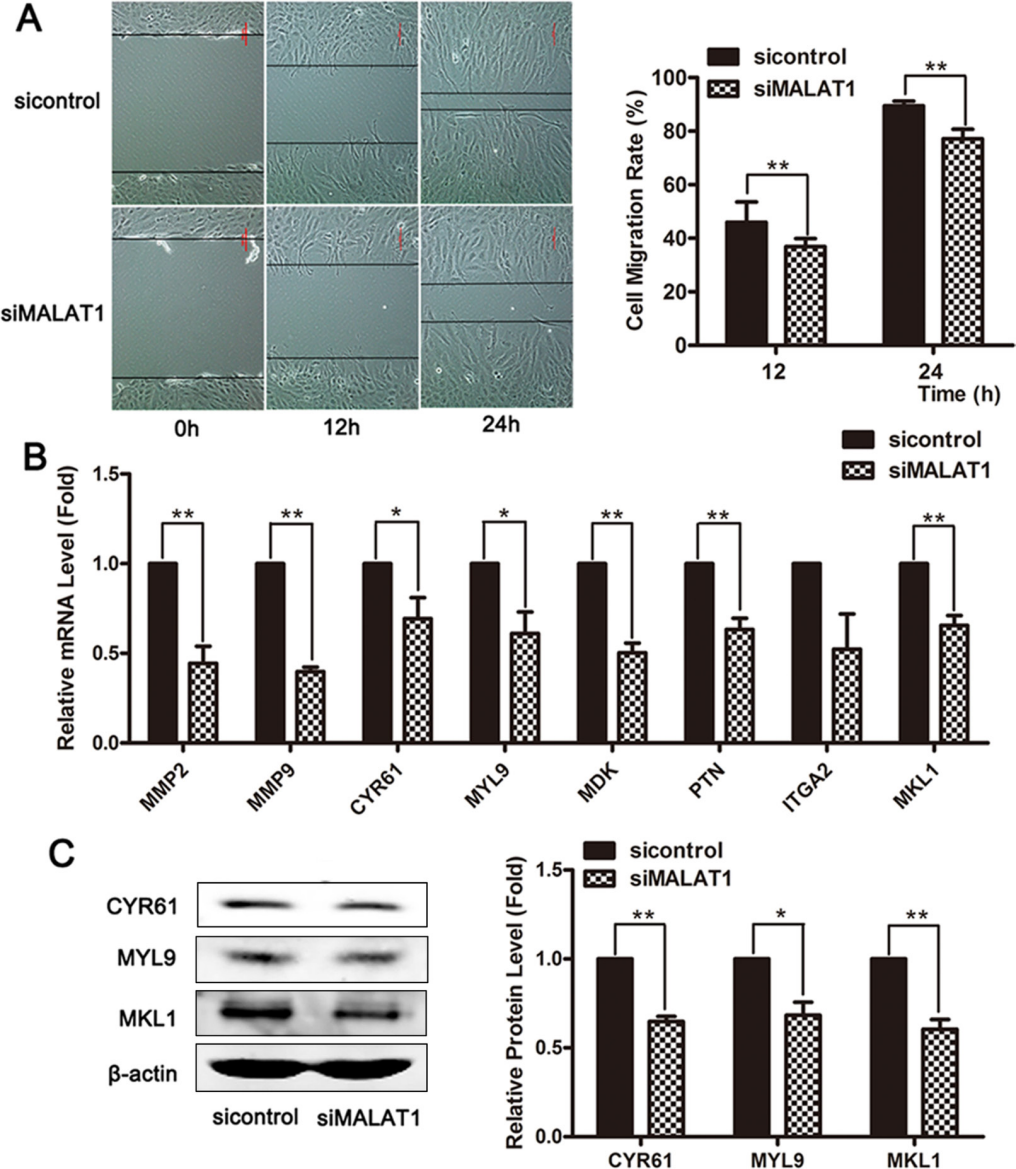
MALAT1 knockdown attenuated the migration of VSMCs (**A**) The cellular migration of VSMCs was evaluated using a wound-healing assay after treatment with MALAT1-specific siRNA. Twenty-four h after transfection with siMALAT1 or control siRNA, the mRNA and protein levels of the migratory markers were subsequently estimated using qRT-PCR (**B**) and western blotting (**C**). ^*^P < 0.05; ^**^P < 0.01.

To investigate the effects of MALAT1 on smooth muscle cell migration, the cellular migration capacity of VSMCs was evaluated after treatment with MALAT1-specific siRNA. Wound-healing assays showed that MALAT1 knockdown inhibited cell migration in VSMCs (Figure 3A). The mRNA levels of multiple migration marker genes were significantly decreased in smooth muscle cells transfected with siMALAT1 compared with the control group (Figure 3B). Western blotting revealed that the downregulation of MALAT1 decreased the expression of migration marker genes (Figure 3C). These results showed that MALAT1 knockdown attenuated the migration of smooth muscle cells.

### BMPs effectively induced systolic phenotype in VSMCs and up-regulated contractile gene expression

**Figure 4:**
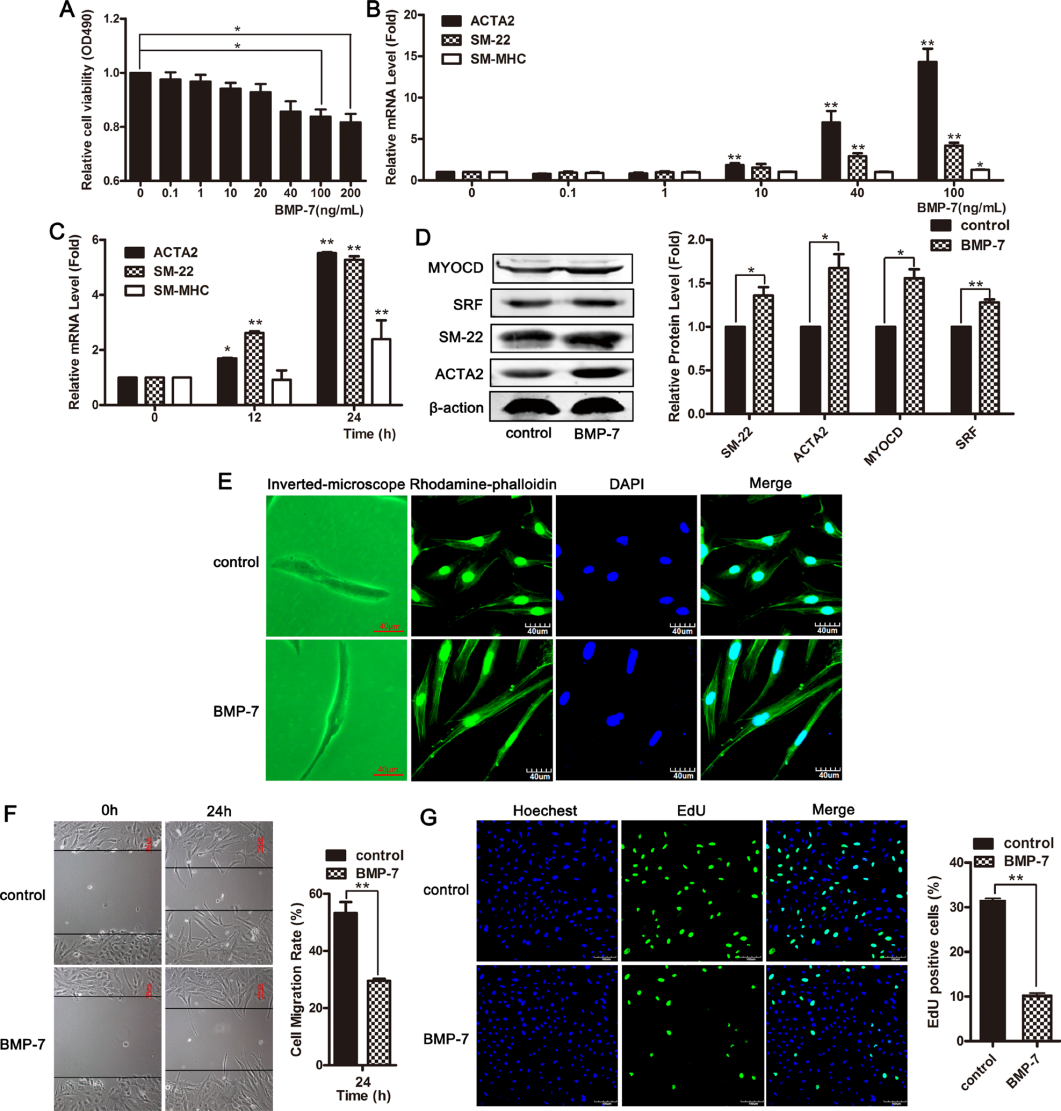
BMPs effectively induced systolic phenotype in VSMCs and up-regulated contractile gene expression (**A**) VSMCs were treated with different concentrations of BMP-7 for 24 h, and MTT assay was used to detect cellular viability. ^*^P < 0.05. (**B**) VSMCs were treated with different concentrations of BMP-7 for 24 h, and the expression of ACTA2, SM-22 and SM-MHC was detected using qRT-PCR. ^*^P <0.05; ^**^P < 0.01. (**C**) The expression of ACTA2, SM-22 and SM-MHC was examined using qRT-PCR on the cells exposed to 100 ng/mL of BMP-7 for 0, 12 and 24 h. ^*^P < 0.05; ^**^P < 0.01. (**D**) Western blotting was performed to detect the expression of ACTA2, SM-22, myocardin and SRF in cells exposed to 100 ng/mL of BMP-7 for 24 h. VSMCs were treated with 100 ng/mL of BMP-7 for 24 h. The morphological changes were observed using phase contrast microscopy (left channel) and confocal microscopy (stained with phalloidin, right channel) (**E**). Cell migration was measured using wound-healing assay (**F**). Cell proliferation was examined using EdU staining (**G**).

We examined the effect of different concentrations of BMP-7 on smooth muscle cell viability using an MTT assay. The data showed that the viability of VSMCs was significantly decreased in response to 100 and 200 ng/mL of BMP-7, and the inhibitory effect of BMP-7 on the proliferation of smooth muscle cells was concentration dependent (Figure 4A). BMP-7 upregulated the mRNA level of contractile genes ACTA2, SM-22, and SM-MHC in concentration and time-dependent manners (Figure 4B and 4C). Stimulation with 100 ng/mL of BMP-7 in VSMCs for 24 h induced a significant upregulation of the expression of ACTA2, SM-22, SRF and myocardin (Figure 4D). We used a combination of phalloidin staining with confocal scanning laser microscopy to observe the cellular morphology at 24 h after BMP-7 treatment. The images showed that smooth muscle cells stimulated with BMP-7 exhibited differentiated phenotypes (Figure 4E). A wound-healing assay showed that BMP-7 inhibited cellular migration in VSMCs (Figure 4F). After treatment with BMP-7 for 24 h, the number of EdU-positive cells was significantly decreased, and cellular proliferation was inhibited, as confirmed using EDU assay (Figure 4G). These data showed that BMPs effectively induced a systolic phenotype in VSMCs through the upregulation of contractile gene expression.

### PDGF-BB induced phenotypic switching in VSMCs from the contractile state to the proliferative state

**Figure 5:**
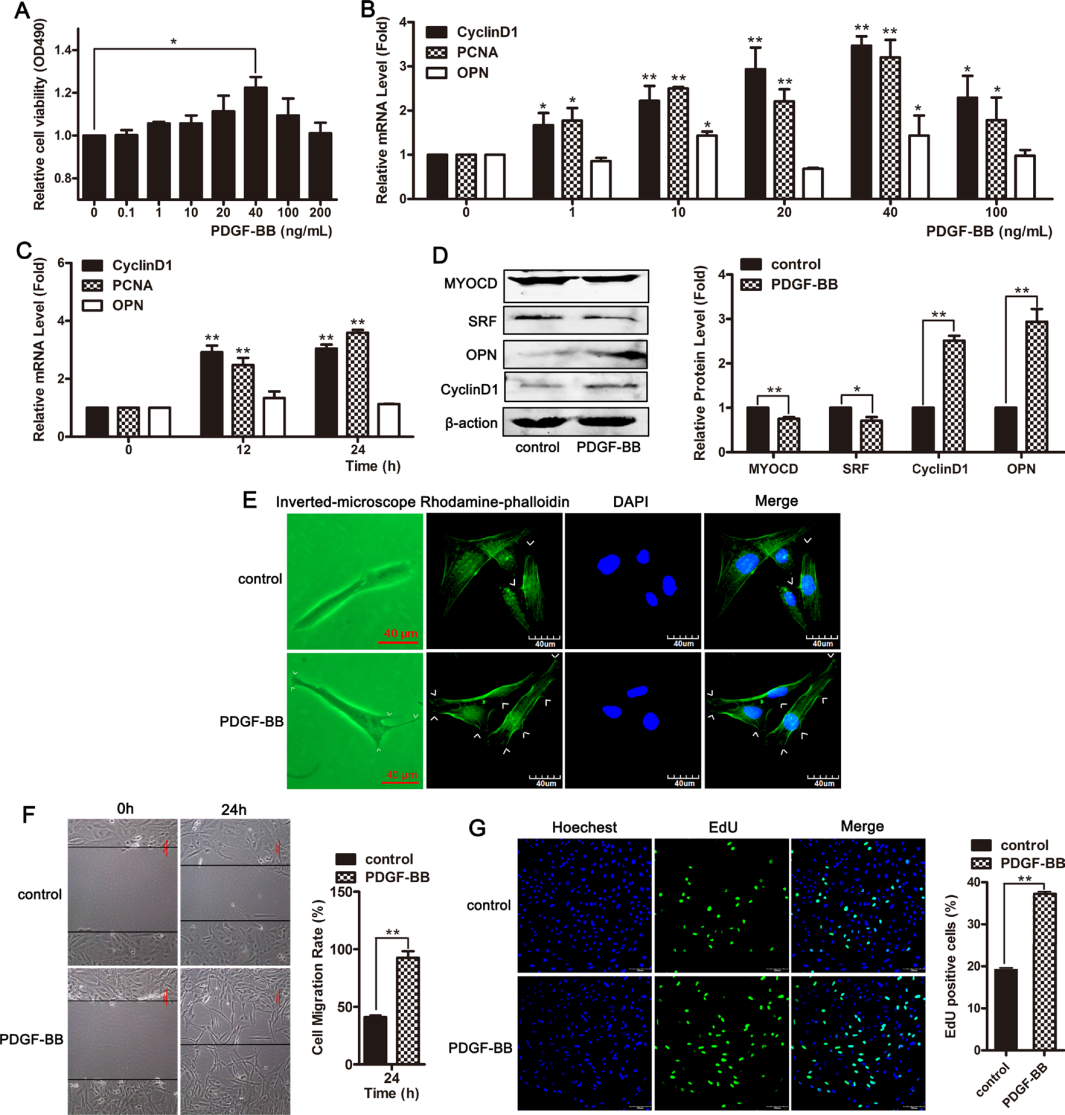
PDGF-BB induced phenotypic switching in VSMCs from the contractile state to the proliferative state (**A**) VSMCs were treated with different concentrations of PDGF-BB for 24 h and subsequently MTT assay was performed to detect cell viability. ^*^P <0.05. (**B**) qRT-PCR was used to detect the mRNA level of PCNA, CyclinD1 and OPN in cells treated with different concentrations of PDGF-BB. ^*^P < 0.05; ^**^P < 0.01. (**C**) The level of PCNA, CyclinD1 and OPN were examined using qRT-PCR in cells exposed to 40 ng/mL of PDGF-BB for 0, 12 and 24 h. ^*^P < 0.05; ^**^P < 0.01.(**D**) Western blotting was performed to detect the expression of ACTA2, SM-22, myocardin and SRF in cells exposed to 40 ng/mL of PDGF-BB for 24 h. VSMCs were treated with 40 ng/mL of PDGF-BB for 24 h. The morphological changes were observed using phase contrast microscopy (left channel) and confocal microscopy (stained with phalloidin, right channel) (**E**). Cell migration was measured using a wound-healing assay (**F**). Cell proliferation was examined using EdU staining (**G**).

VSMCs were treated with different concentrations of PDGF-BB for 24 h, and subsequently, an MTT assay was performed to detect the cell viability. As shown in Figure 5A, the viability of VSMCs was increased in response to treatment with 40 ng/mL of PDGF-BB. The expression of proliferative marker genes cyclin D1 and PCNA was significantly increased in PDGF-BB-treated VSMCs (Figure 5B and 5C). PDGF-BB promoted cellular proliferation in concentration- and time-dependent manners (Figure 5A–5C). PDGF-BB significantly upregulated the expression of the proliferation markers CyclinD1 and OPN in HVSMCs and downregulated the expression of differentiation markers myocardin and SRF (Figure 5D). At 24 h after treatment with 40 ng/mL of PDGF-BB, the cell morphology and cytoskeleton were observed. Compared to the control group, smooth muscle cells treated with PDGF-BB exhibited a proliferative phenotype, and the cells were fibrous with extended pseudopods (Figure 5E). Wound-healing and EdU assays revealed that PDGF-BB promoted cell migration and proliferation (Figure 5F and 5G). These results showed that PDGF-BB induced phenotypic switching in VSMCs from the contractile to the proliferative state.

### MALAT1 increased PDGF-BB-induced proliferation and migration of VSMCs

**Figure 6:**
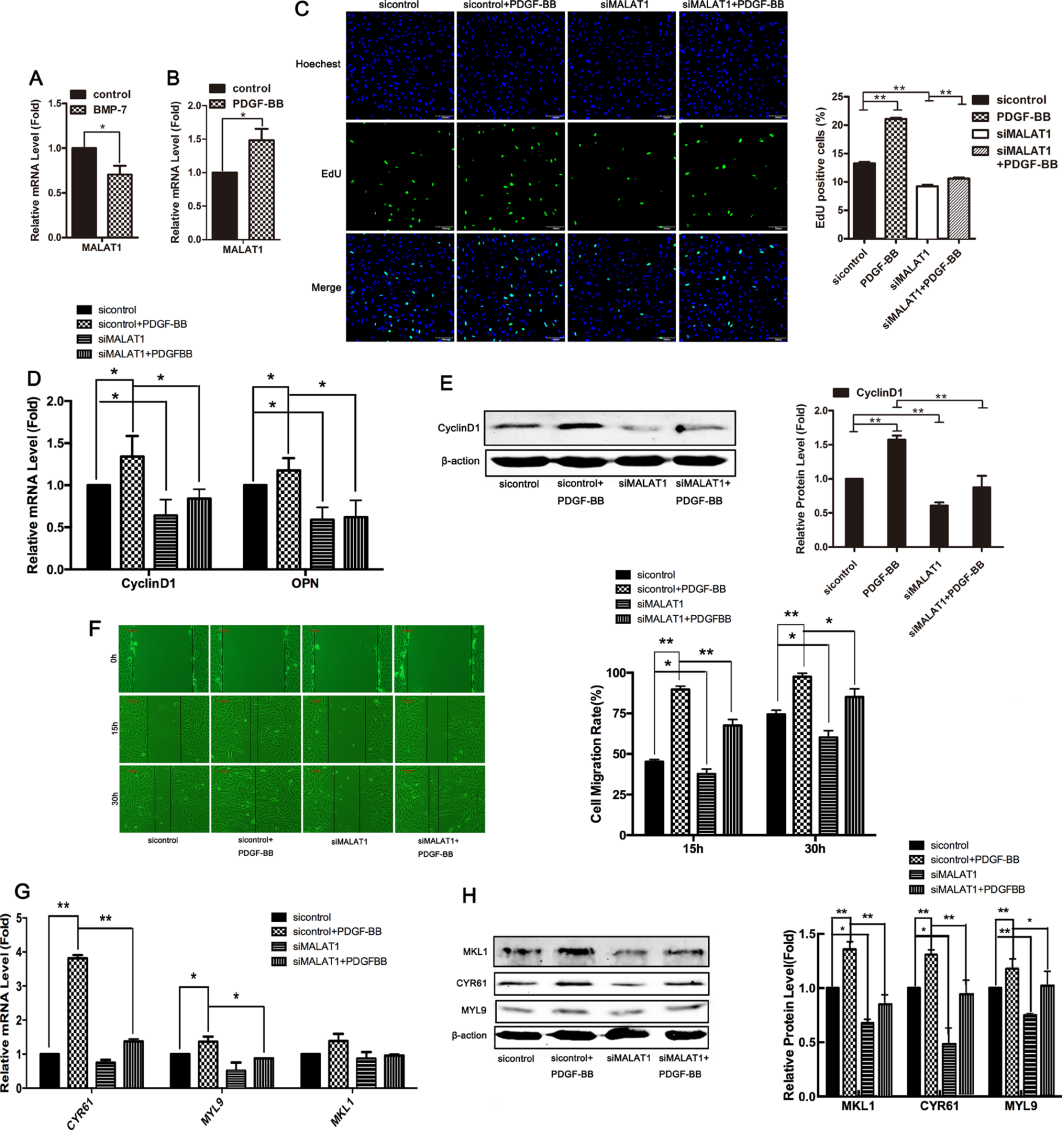
MALAT1 increased PDGF-BB-induced proliferation and migration of VSMCs The level of MALAT1 was examined in HVSMCs treated with 100 ng/mL of BMP-7 (**A**) or 40 ng/mL of PDGF-BB (**B**) using qRT-PCR. Twenty-four h after transfection with siMALAT1 or sicontrol, cells were stimulated with PDGF-BB for another 24 h. The proliferation ability of SMC was evaluated using EdU staining (**C**). The mRNA level of Cyclin D1 and OPN was detected using qRT-PCR (**D**). ^*^P < 0.05. Western blotting was performed to test the expression of CyclinD1 (**E**). Wound-healing assay were carried out to measure cell migration (**F**). The level of CYR61, MYL9 and MKL1was examined using qRT-PCR (**G**) and western blotting (**H**). ^*^P < 0.05; ^**^P < 0.01.

To further investigate the role of MALAT1 in the phenotype switching of smooth muscle cells, the level of MALAT1 in VSMCs treated with BMP-7 or PDGF-BB was examined using qRT-PCR. The results showed that BMP-7, which induced the differentiation of VSMCs, attenuated the level of MALAT1, while PDGF-BB, which induced the proliferation of smooth muscle cells, enhanced the expression of MALAT1 (Figure 6A and 6B).

To support the notion that MALAT1 is required for the PDGF-BB-induced proliferation of VSMCs, we examined whether the downregulation of MALAT1 attenuates PDGF-BB-induced proliferation and migration. 24 h after transfection with siMALAT1 or sicontrol, the cells were stimulated with PDGF-BB for another 24 h. As shown in Figure 6C–6E, PDGF-BB induced cell proliferation and upregulated the expression of Cyclin D1 and OPN, while knockdown of MALAT1 inhibited PDGF-BB-induced proliferation and attenuated the PDGF-BB-induced expression of proliferative genes. Next, we investigated whether MALAT1 enhanced the PDGF-BB-induced migration of VSMCs. Compared with the control group, the migration rate of cells treated with PDGF-BB was significantly increased (Figure 6F). Knockdown of MALAT1 inhibited the migration of cells stimulated by PDGF-BB (Figure 6F). MALAT1 knockdown inhibited the PDGF-BB-induced activation of the migratory genes CYR61, MYL9 and MKL1 at both the mRNA and protein levels (Figure 6G and 6H). These results suggest that MALAT1 was required for the PDGF-BB-induced proliferation and migration of smooth muscle cells.

### MALAT1 acts as ceRNA to enhance ATG7 expression by sponging miR142-3p

**Figure 7:**
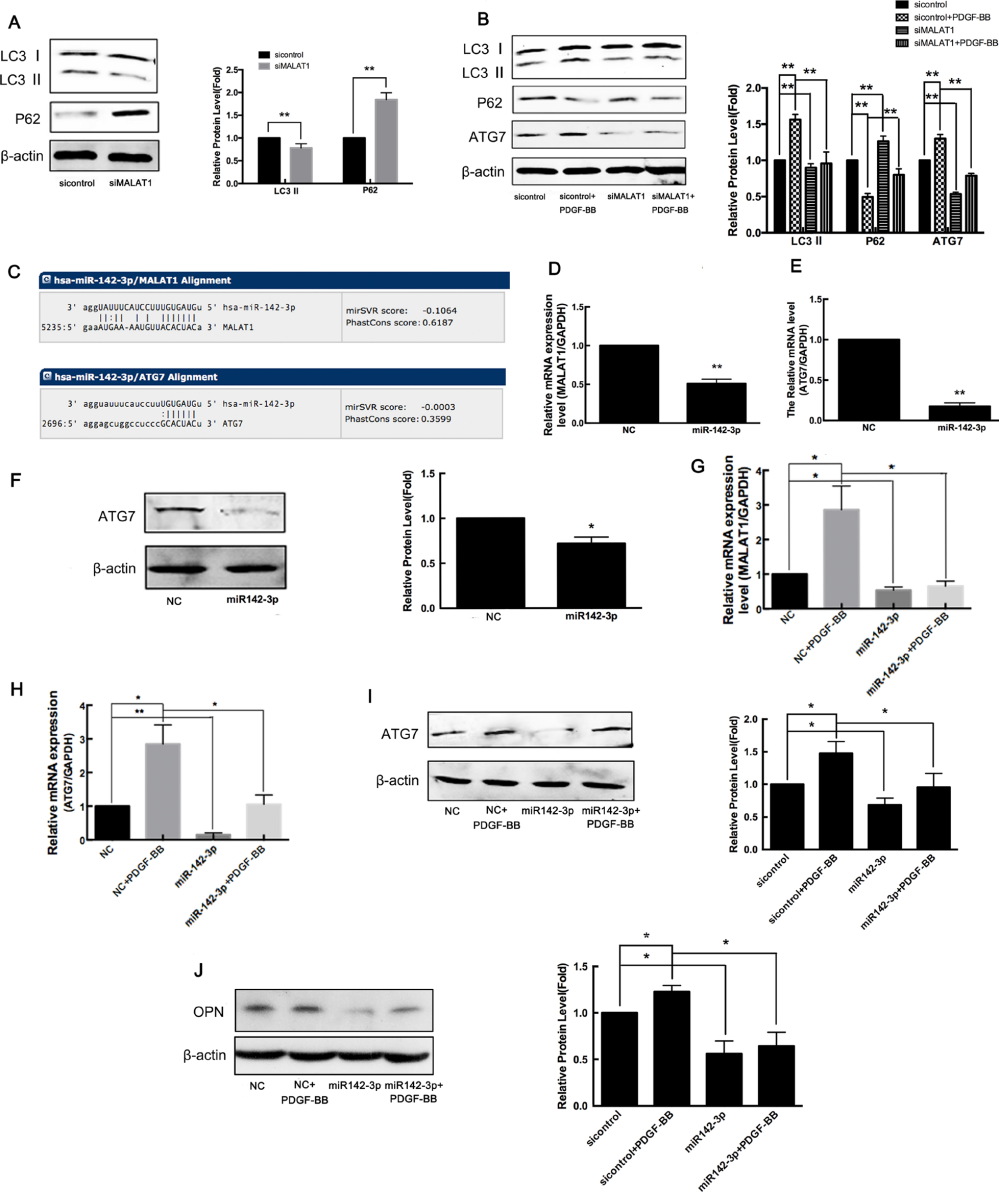
MALAT1 acts as ceRNA to enhance ATG7 expression by sponging miR142-3p (**A**) VSMCs were transfected with siMALAT1 for 24 h and subsequently the expression of LC3-I/LC3-II and p62 was detected using western blotting. (**B**) Twenty-four h after transfection with siMALAT1 or sicontrol, the cells were stimulated with PDGF-BB for another 24 h, and subsequently the expression of LC3-I/LC3-II, p62 and ATG7 was detected using western blotting. (**C**) According to the miRNA databases, miR142-3p is a potential miRNA target of MALAT1 and autophagy gene ATG7 is a potential target gene of miR142-3p. (**D**) The miR142-3p mimics were transfected into HVSMCs for 24 h, and subsequently the level of MALAT1 was detected using qRT-PCR. ^*^P < 0.05. The miR142-3p mimics were transfected into VSMCs for 24 h, and subsequently the expression of ATG-7 detected using qRT-PCR (**E**) and western blotting (**F**). ^**^P < 0.01. The miR142-3p mimics were transfected into VSMCs in the presence of PDGF-BB for 24 h. The level of MALAT1 was detected using qRT-PCR (**G**). ^*^P < 0.05. The expression of ATG-7 was detected using qRT-PCR (**H**) and western blotting (**I**). ^*^P < 0.05. The expression of OPN was detected using western blotting (**J**). ^*^P < 0.05.

The PDGF-induced transformation of smooth muscle cells from contraction to synthetic phenotypes is accompanied by an increase in autophagy. Consistent with previous studies [36], the results of the present study also showed that treatment with PDGF-BB for 24 h induced the activation of autophagy in VSMCs, consistent with the decrease of P62 expression and increase of LC3-II and ATG7 expression (Figure 7B). To investigate whether MALAT1 activates autophagy in VSMCs, the cells were transfected with siMALAT1 for 24 h, and subsequently, the expression of LC3-I/LC3-II and p62 was detected using western blotting. The results showed that MALAT1 knockdown decreased LC3-II expression and increased p62 expression, indicating the attenuation of autophagy (Figure 7A). As shown in Figure 6, MALAT1 was increased in VSMCs in response to PDGF-BB. Thus, we explored whether MALAT1 was involved in regulating the autophagy in PDGF-BB-induced proliferation of smooth muscle cells. Downregulation of MALAT1 significantly inhibited the autophagy induced through PDGF-BB (Figure 7B).

LncRNAs function as competing endogenous RNAs (ceRNAs) to regulate gene expression by sponging target microRNA [37]. Thus, we employed miRNA databases (http://www.microrna.org/) to search for potential miRNA recognition elements on the lncRNA MALAT1. Using miRNA databases, miR142-3p was predicted as a potential miRNA target of MALAT1, and autophagy gene ATG7 was predicted as a potential target gene of miR142-3p (Figure 7C). To confirm whether MALAT1 acts as a ceRNA to regulate ATG7 expression by sponging miR142-3p, miR142-3p mimics were transfected into VSMCs for 24 h, and subsequently the level of MALAT1 and the expression of ATG-7 were detected using qRT-PCR and western blotting, respectively. 24 h after transfection with miR-142-3p mimics, the level of MALAT1 was significantly downregulated, and the expression of ATG7 was also decreased (Figure 7D–7F). To explore the role of miR-142-3p in PDGF-activated autophagy, miR142-3p mimics were transfected into VSMCs in the presence of PDGF-BB for 24 h. As shown in Figure 7G, miR-142-3p inhibited the PDGF-BB-induced increase of MALAT1 levels. PDGF-BB enhanced the expression of ATG7, while transfection with miR-142-3p mimics attenuated the upregulation of ATG7 in response to PDGF-BB (Figure 7H and 7I). PDGF-BB enhanced the expression of the proliferating cell marker OPN, whereas transfection with miR-142-3p mimics attenuated the upregulation of OPN in response to PDGF-BB, which inhibited the transformation of smooth muscle cells to a proliferative phenotype (Figure 7J). These results showed that MALAT1 acted as miR142-3p sponge to enhance ATG7 expression, leading to the activation of autophagy and the switch of smooth muscle cells to a proliferative phenotype.

## DISCUSSION

MALAT1 was initially identified as a marker for early non-small cell lung cancer. The expression of MALAT1 increased during tumorigenesis and metastasis and promoted the occurrence, proliferation and migration of epithelial-mesenchymal transition (EMT) in tumor cells [38]. MALAT1 also promotes the development of EMT in retinal pigment cells, suggesting that MALAT1 plays an important role in fibrosis. Subsequently, studies have demonstrated that MALAT1 is also highly expressed in a variety of normal organs [39]. MALAT1 exerts an effect on vascular endothelial dysfunction. MALAT1 is highly expressed in endothelial cells, and its expression under hypoxia conditions significantly promoted the proliferation of endothelial cells. MALAT1 effectively improves blood flow recovery and capillary density after hind limb ischemia, suggesting that MALAT1 affects angiogenesis in ischemic hearts [40]. Studies have shown that MALAT1 in hypoxic pulmonary arterial smooth muscle cells in the expression level was significantly increased. Smooth muscle cells play a vital role in cardiovascular disease. Here, we observed that MALAT1 was involved in the phenotypic switch of VSMCs. Knockdown of MALAT1 promoted the transformation of smooth muscle cells from a proliferative phenotype to a differentiated phenotype, consistent with the observed increase of the expression of contractile genes and inhibition of the expression of proliferative and migrated genes. MALAT1 knockdown also inhibited cellular proliferation and migration, as demonstrated using MTT, EdU staining, and wound healing assays. Consistently, Brock *et al*. reported that MALAT1 silencing significantly reduced the proliferation and migration of human pulmonary artery smooth muscle cells [41]. Furthermore, the results of the present study data showed that knocking down MALAT1 in VSMCs led to significant cell cycle arrest in the G2 phase. Yang *et al*. observed that MALAT1 transcripts, predominately localized in the nucleus, are translocated to the cytoplasm during the G2/M cell cycle phase. Silencing the expression of MALAT-1 leads to G2/M arrest in HeLa cells [42].

Although lncRNAs regulate a variety of cellular functions, several reports have described their effects on the phenotype switching of VSMCs. LncRNA Growth Block Specificity 5 (GAS5) has been identified as a novel modulator of SMC affecting Smad3 function in differentiation. GAS5 binds to Smad3 through its rSBE and disrupts the binding of Smad3 to SBE DNA in the promoter of the SMC differentiation gene. Reflecting this competitive combination, GAS5 negatively regulates TGF-β/Smad3 signaling, thereby inhibiting TGF-β-induced SMC differentiation [43]. Smooth muscle and endothelial cell enriched migration/differentiation-associated long non-coding RNA (SENCR) is another lncRNA recently identified from human coronary artery SMCs. The knockdown of SENCR decreased the expression of myocardin and contractile genes and increased the expression of promigratory genes [44]. The results of the present study showed that MALAT1 was downregulated in BMP-7-induced VSMC differentiation and upregulated in PDGF-BB-induced cellular proliferation. The downregulation of MALAT1 attenuated the PDGF-BB-induced proliferation and migration of smooth muscle cells.

PDGF binds to surface receptors and activates several intracellular signaling pathways that ultimately regulate gene expression and cellular function, thereby decreasing the expression of contractile proteins and increasing the expression of synthetic proteins, such as osteopontin and vimentin. Studies have shown that autophagy is activated in VSMCs to remove proteins affected by oxidative damage [45], as these proteins form large aggregates that may be particularly difficult to proteolyze. Consistent with other reports, the results of the present study also confirmed that the PDGF-induced transformation from a contraction to a synthetic phenotype is associated with an increase in autophagy, and autophagy plays a crucial role in the conversion of VSMCs from a contractile to a synthetic phenotype.

Recent studies have reported that Malat1 inhibits apoptosis by stimulating autophagy [46]. MALAT1 induces autophagy in tumor cells under hypoxia and stabilizes HIF-1a in human liver L-02 cells [47, 48]. Functionally, MALAT1 promotes the development of cancer through a variety of mechanisms that regulate cancer cell radiosensitivity and chemical sensitivity, such as miRNA sponging, enhanced EMT and stimulated autophagy [46, 49, 50]. Here, we reported that the downregulation of MALAT1 significantly inhibited the basal autophagy level in VSMCs. Knocking down MALAT1 also significantly attenuated PDGF-BB-induced autophagy. Taken together with previous studies [46–51], MALAT1 plays an important role in stimulating autophagy.

LncRNAs may act as competing endogenous RNAs (ceRNAs), namely miRNA sponges or antagomirs, which interact with miRNAs and regulate the expression of miRNA target proteins [51, 52]. Here, we observed that miR142-3p decreased the expression of MALAT1 and ATG7. Recently, an increasing number of miRNAs, such as miR-20a, miR-137 and miR-17, have been demonstrated to inhibit the autophagic process by targeting ATG7 [53]. We first identified a new miRNA, miR142-3p, which targets ATG7. MALAT1 acts as a miR142-3p sponge to regulate ATG7 expression, leading to the activation of autophagy and the switch of smooth muscle cells to a proliferative phenotype. Thus, the downregulated level of MALAT1 in BMP-7-treated VSMCs resulted in the increase of miR142-3p, and then miR142-3p inhibited the expression of ATG7 and autophagy level by binding to the 3′UTR of ATG7. In conclusion, the present study reveals a novel mechanism by which MALAT1 promotes the transformation of smooth muscle cells from contraction to synthetic phenotypes.

## MATERIALS AND METHODS

### Cell culture and treatment

The cell lines used in the present study were all obtained from the American Type Culture Collection (ATCC, Manassas, VA). VSMCs were cultured in Dulbecco's modified high glucose medium (DMEM-HG, HyClone, United States) supplemented with 10% fetal bovine serum (FBS, Sijiqing, China) and 1% penicillin-streptomycin-amphotericin B (Sangon Biotech, China) at 37°C in a humidified incubator (Thermo Forma, United States) containing 5% CO2.

VSMCs were seeded onto 6-well plates at 1 × 10^6^ cells/mL. The cells were starved in serum-free media for another 24 h and subsequently treated with 40 ng/mL of PDGF-BB or 100 ng/mL of BMP-7.

### Cell transfection

For short-term depletion experiments, the following siRNA sequences were used: siMALAT1 (F: 5′-CUAGAAUCCUAAAGGCAAAdTdT-3′ and R: 5′-UUUGCCUUUAGGAUUCUAGUAGdTdT-3′) (Invitrogen, United States); control siRNA (F:5′-UUCUUCGAA CGUGUCACGUTT-3′ and R: 5′-ACGUGACACGUUCG GAGAATT-3′) (Invitrogen, United States). VSMCs was transfected with siRNA oligonucleotides using HiPerFect (Invitrogen, United States) according to the manufacturer's instructions. Subsequently, phalloidin staining, EDU staining, cell migration assays, PCR and western blotting were performed as described.

### Quantitative real-time RT-PCR (qRT-PCR)

For qRT-PCR analysis, total RNA was extracted using TRIzol reagent (Ambion, United States), according to the manufacturer's instructions. The RNA (2 μg of each sample) was reverse-transcribed into cDNA using random primers (Invitrogen, United States) and M-MLV reverse transcriptase (Promega, China) according to the manufacturer's instructions. The qRT-PCR was performed using an Applied

Biosystems StepOne QPCR System. Fast SYBR^®^Green Master Mix was obtained from Applied Biosystems. The data are shown as the relative expression level normalized to Glyceral-dehyde-3-phosphate dehydrogenase (GAPDH). The qRT-PCR primer sequences used in the present study are shown in Table 1.

**Table 1:** Primers for real-time PCR

Gene	Primer sequence (5′→3′)
GAPDH	F- ATTCAACGGCACAGTCAAGG
	R- GCAGAAGGGGCGGAGATGA
MALAT1	F-GTGATGCGAGTTGTTCTCCG
	R-CTGGCTGCCTCAATGCCTAC
ACTA2	F-AGCCAAGCACTGTCAGGAATC
	R-GAGCCCAGAGCCATTGTCAC
SM-22	F-AGCAAGCTGGTGAACAGCCTGTACC
	R-ATCATTCTTGGTCACTGCCAAGCTG
PCNA	F-GGCTCCATCCTCAAGAAGGTGTT
	R-CGTTATCTTCGGCCCTTAGTGTA
CyclinD1	F-GCTGTGCATCTACACCGACAACTC
	R-TTGCGGATGATCTGTTTGTTCTCCT
OPN	F-AGTACCCTGATGCTACAGACGAG
	R-CGTTTCATAACTGTCCTTCCCAC
CYR61	F-AAGGATAGTATCAAGGACCCC
	R-ATCCATTCCAAAAACAGGGAG
MYL9	F-CAGTCCCAGATCCAGGAGTTTAAG
	R-GGGTGAACTCCACGTAGTTGAAGT
MKL1	F-CATCTGTCTGTGGGAATTGTAAGC
	R-ACACGAGCACTGGTTCATCATC
Myocardin	F-AGTAAGAACCGCCACAAA
	R-GAGCATAGGCAGAGTCCA
SRF	F-AATGAGTGCCACTGGCTTTGAAGA
	R-TGGAGGTTGTACCCGGCAGGTTGG
ATG7	F-GTGTACGATCCCTGTAACCTAACCC
	R-CGAAAGCAGAGAACTTCAACAGACT
miR142-3p	F-ACACTCCAGCTGGCTGTAGTGTTTCCTACT
	R- CTCAACTGGTGTCGTGGA
U6	F- GCTTCGGCAGCACATATACTAAAAT
	R- CGCTTCACGAATTTGCGTGTCAT

### Phalloidin staining

The cells were seeded onto 24-well plates at 0.25 × 10^6^ cells/mL and cultured to 60–70% confluency. After transfection or/and treatment, the cells were washed with 0.1% Triton X-100 for 5 minutes and then washed with phosphate-buffered saline (PBS). The cells were washed with 1% BSA at room temperature for 20 minutes and subsequently incubated with 5 μg/mL of FITC-phalloidin in a 37°C incubator for 1 h, follow by DAPI staining for 5 minutes. Subsequently, the cells were observed and photographed using laser confocal microscopy (OLYMPUS, Japan).

### Immunofluoresence staining

Immunocytochemistry staining has been previously described [54]. Briefly, the cells were fixed in 4% paraformaldehyde for 20 minutes and subsequently blocked with conventional goat serum at room temperature for 1 hour. After incubating with rabbit anti-human ACTA2 (Santa Cruz, United States) antibody overnight at 4°C, the cells were stained with fluorescein isothiocyanate (FITC)-labeled anti-rabbit secondary antibody, and 4', 6-diamidino-2-phenylindole (DAPI) was used to stain the nucleus. The images were captured using a laser scanning confocal microscope (OLYMPUS, Japan). The average optical density of immunofluorescence was analyzed by Image J software.

### Western blotting

Western blotting was performed as previously described [54]. After incubation with the primary antibodies, including anti-rabbit Myocardin (Sigma, United States), anti-rabbit MRTF-A (Abcam, United States), anti-rabbit SRF (Santa Cruz, United States), anti-rabbit ACTA2 (Abcam, United States), anti-rabbit SM-22 (Abcam, United States), anti-rabbit OPN (Santa Cruz, United States), anti-rabbit CyclinD1 (Santa Cruz, United States), anti-Rabbit CYR61 (Santa Cruz, United States), anti-rabbit MYL9 (Santa Cruz, United States), anti-rabbit LC3 (Novus Biologicals, United States), anti-mouse P62 (BD Transduction Laboratories, United States), anti-rabbit ATG7 (Bioss, China) and anti-mouse β-actin (Santa Cruz, United States) antibodies; the membranes were incubated with the appropriate secondary antibodies, including IR Dye-800 conjugated anti-rabbit IgG secondary antibody and IR Dye-680 conjugated anti-mouse IgG. The specific protein was visualized using the Odyssey Infrared Imaging System (Gene Company, HongKong). β-actin expression was used as an internal control to show equal loading of the protein sample. The relative quantity of proteins was analyzed using Image J software. The relative quantity of proteins was analyzed using Image J software.

### Cell viability assay

HA-VSMCs were seeded onto 96-well plates and subsequently transfected with siMALAT1 or treated with PDGF-BB or BMP. The 3-(4,5-dimethylthiazol-2-yl)-2,5-diphenyltetrazole (MTT) assay was performed to measure cell viability. For this assay, 20 μL of MTT dye (Solarbio, United States) was added to each well, followed by incubation for 4 h. The absorbance was measured at 490 nm or 570 nm using a multifunctional microplate reader (Infinite M200 PRO, Tecan, Switzerland).

### EdU staining

EdU staining was performed using the EdU kit (Ribobio, China) according to the manufacturer's instructions. Briefly, the cells were seeded onto 96-well plates and subsequently exposed to the indicated treatments, accordingly. The cells were incubated in 100 μL of medium containing 50 μM EdU for 2 h and subsequently fixed with 4% paraformaldehyde, followed by staining with Hoechst 33342. The samples were observed under a laser confocal microscope (OLYMPUS, Japan).

### Flow cytometry

For cell cycle analysis, HVSMCs were transfected with siMALAT1 or sicontrol for 24 h and subsequently fixed in 75% alcohol. After washing with PBS, the cells were incubated in 50 mg/mL of propidium iodide (PI) containing 0.25% Triton X-100 for 2 h. The cells were analyzed using a FACScan flow cytometer (Accuri C6, BD Bioscience, United States), and the cell cycle distribution was calculated using Modfit software.

### Wound-healing assay

HA-VSMCs were seeded onto 6-well plates and subsequently transfected with siMALAT1 or control siRNA for 6 h. Six h after transfection, the cell monolayer was scratched with a 10 μL pipette tip and sequentially cultured at 37°C in a 5% CO_2_ incubator chamber. Images were captured at zero, 12 and 24 h after wounding using an inverted phase contrast microscope.

### Statistical analysis

Data were expressed as the means ± SE, accompanied by the number of experiments independently performed, and subsequently analyzed using a *t*-test. Differences with *P <* 0.05 were considered statistically significant.

## References

[1] Arimura C, Suzuki T, Yanagisawa M, Imamura M, Hamada Y, Masaki T (1988). Primary structure of chicken skeletal muscle and fibroblast alpha-actinins deduced from cDNA sequences. Eur J Biochem.

[2] Babij P, Kelly C, Periasamy M (1991). Characterization of a mammalian smooth muscle myosin heavy-chain gene: complete nucleotide and protein coding sequence and analysis of the 5′ end of the gene. Proc Natl Acad Sci USA.

[3] Madsen CS, Regan CP, Hungerford JE, White SL, Manabe I, Owens GK (1998). Smooth muscle-specific expression of the smooth muscle myosin heavy chain gene in transgenic mice requires 5′-flanking and first intronic DNA sequence. Circ Res.

[4] Miano JM, Cserjesi P, Ligon KL, Periasamy M, Olson EN (1994). Smooth muscle myosin heavy chain exclusively marks the smooth muscle lineage during mouse embryogenesis. Circ Res.

[5] Duband JL, Gimona M, Scatena M, Sartore S, Small JV (1993). Calponin and SM 22 as differentiation markers of smooth muscle: spatiotemporal distribution during avian embryonic development. Differentiation.

[6] Miano JM, Carlson MJ, Spencer JA, Misra RP (2000). Serum response factor-dependent regulation of the smooth muscle calponin gene. J Biol Chem.

[7] Kim S, Ip HS, Lu MM, Clendenin C, Parmacek MS (1997). A serum response factor-dependent transcriptional regulatory program identifies distinct smooth muscle cell sublineages. Mol Cell Biol.

[8] van der Loop FT, Schaart G, Timmer ED, Ramaekers FC, van Eys GJ (1996). Smoothelin, a novel cytoskeletal protein specific for smooth muscle cells. J Cell Biol.

[9] Dzau VJ, Braun-Dullaeus RC, Sedding DG (2002). Vascular proliferation and atherosclerosis: new perspectives and therapeutic strategies. Nat Med.

[10] Dzau VJ, Gibbons GH (1987). Autocrine-paracrine mechanisms of vascular myocytes in systemic hypertension. Am J Cardiol.

[11] Heldin CH, Westermark B (1999). Mechanism of action and *in vivo* role of platelet-derived growth factor. Physiol Rev.

[12] Ross R (1999). Atherosclerosis—an inflammatory disease. N Engl J Med.

[13] Barrett TB, Benditt EP (1987). sis (platelet-derived growth factor B chain) gene transcript levels are elevated in human atherosclerotic lesions compared to normal artery. Proc Natl Acad Sci USA.

[14] Rubin K, Tingström A, Hansson GK, Larsson E, Rönnstrand L, Klareskog L, Claesson-Welsh L, Heldin CH, Fellström B, Terracio L (1988). Induction of B-type receptors for platelet-derived growth factor in vascular inflammation: possible implications for development of vascular proliferative lesions. Lancet.

[15] Tanizawa S, Ueda M, van der Loos CM, van der Wal AC, Becker AE (1996). Expression of platelet derived growth factor B chain and beta receptor in human coronary arteries after percutaneous transluminal coronary angioplasty: an immunohistochemical study. Heart.

[16] Ueda M, Becker AE, Kasayuki N, Kojima A, Morita Y, Tanaka S (1996). In situ detection of platelet-derived growth factor-A and -B chain mRNA in human coronary arteries after percutaneous transluminal coronary angioplasty. Am J Pathol.

[17] Attisano L, Wrana JL (2002). Signal transduction by the TGF-beta superfamily. Science.

[18] Heldin CH, Miyazono K, ten Dijke P (1997). TGF-beta signalling from cell membrane to nucleus through SMAD proteins. Nature.

[19] Massagué J (1998). TGF-beta signal transduction. Annu Rev Biochem.

[20] Zhang S, Fantozzi I, Tigno DD, Yi ES, Platoshyn O, Thistlethwaite PA, Kriett JM, Yung G, Rubin LJ, Yuan JX (2003). Bone morphogenetic proteins induce apoptosis in human pulmonary vascular smooth muscle cells. Am J Physiol Lung Cell Mol Physiol.

[21] Lagna G, Nguyen PH, Ni W, Hata A (2006). caspase-9 and caspase-8 mediates apoptosis in pulmonary artery smooth muscle cells. Am J Physiol Lung Cell Mol Physio Physiol. BMP-dependent activation of.

[22] Dorai H, Vukicevic S, Sampath TK (2000). Bone morphogenetic protein-7 (osteogenic protein-1) inhibits smooth muscle cell proliferation and stimulates the expression of markers that are characteristic of SMC phenotype *in vitro*. J Cell Physiol.

[23] Lee J, Giordano S, Zhang J (2012). Autophagy, mitochondria and oxidative stress: cross-talk and redox signalling. Biochem J.

[24] Mercer TR, Dinger ME, Mattick JS (2009). Long non-coding RNAs: insights into functions. Nat Rev Genet.

[25] Yang F, Bi J, Xue X, Zheng L, Zhi K, Hua J, Fang G (2012). Up-regulated long non-coding RNA H19 contributes to proliferation of gastric cancer cells. FEBS J.

[26] Ji P, Diederichs S, Wang W, Böing S, Metzger R, Schneider PM, Tidow N, Brandt B, Buerger H, Bulk E, Thomas M, Berdel WE, Serve H, Müller-Tidow C (2003). MALAT-1, a novel noncoding RNA, and thymosin beta4 predict metastasis and survival in early-stage non-small cell lung cancer. Oncogene.

[27] Hou Z, Xu X, Zhou L, Fu X, Tao S, Zhou J, Tan D, Liu S (2017). The long non-coding RNA MALAT1 promotes the migration and invasion of hepatocellular carcinoma by sponging miR-204 and releasing SIRT1. Tumour Biol.

[28] Wu L, Wang X, Guo Y (2017). Long non-coding RNA MALAT1 is upregulated and involved in cell proliferation, migration and apoptosis in ovarian cancer. Exp Ther Med.

[29] Li J, Gao J, Tian W, Li Y, Zhang J (2017). Long non-coding RNA MALAT1 drives gastric cancer progression by regulating HMGB2 modulating the miR-1297. Cancer Cell Int.

[30] Yin Z, Pascual C, Klionsky DJ (2016). Autophagy: machinery and regulation. Microb Cell.

[31] Xiong J (2015). Atg7 in development and disease: panacea or Pandora's Box?. Protein Cell.

[32] Nakatogawa H (2013). Two ubiquitin-like conjugation systems that mediate membrane formation during autophagy. Essays Biochem.

[33] Uchiyama Y, Shibata M, Koike M, Yoshimura K, Sasaki M (2008). Autophagy-physiology and pathophysiology. Histochem Cell Biol.

[34] Komatsu M, Waguri S, Ueno T, Iwata J, Murata S, Tanida I, Ezaki J, Mizushima N, Ohsumi Y, Uchiyama Y, Kominami E, Tanaka K, Chiba T (2005). Impairment of starvation-induced and constitutive autophagy in Atg7-deficient mice. J Cell Biol.

[35] Li J, Zhao L, Yang T, Zeng YJ, Yang K (2014). C-ski Inhibits Autophagy of Vascular Smooth Muscle Cells Induced by oxLDL and PDG. PLoS One.

[36] Salabei JK, Cummins TD, Singh M, Jones SP, Bhatnagar A, Hill BG (2013). PDGF-mediated autophagy regulates vascular smooth muscle cell phenotype and resistance to oxidative stress. Biochem J.

[37] Han X, Yang F, Cao H, Liang Z (2015). Malat1 regulates serum response factor through miR-133 as a competing endogenous RNA in myogenesis. FASEB J.

[38] Fan Y, Shen B, Tan M, Mu X, Qin Y, Zhang F, Liu Y (2014). TGF-β-induced upregulation of malat1 promotes bladder cancer metastasis by associating with suz12. Clin Cancer Res.

[39] Gutschner T, Hämmerle M, Diederichs S (2013). MALAT1— a paradigm for long noncoding RNA function in cancer. J Mol Med (Berl).

[40] Yang S, Yao H, Li M, Li H, Wang F (2016). Long Non-Coding RNA MALAT1 Mediates Transforming Growth Factor Beta1-Induced Epithelial-Mesenchymal Transition of Retinal Pigment Epithelial Cells. PLoS One.

[41] Brock M, Schuoler C, Leuenberger C, Bühlmann C, Haider TJ, Vogel J, Ulrich S, Gassmann M, Kohler M, Huber LC (2017). Analysis of hypoxia-induced noncoding RNAs reveals metastasis-associated lung adenocarcinoma transcript 1 as an important regulator of vascular smooth muscle cell proliferation. Exp Biol Med (Maywood).

[42] Yang F, Yi F, Han X, Du Q, Liang Z (2013). MALAT-1 interacts with hnRNP C in cell cycle regulation. FEBS Lett.

[43] Tang R, Zhang G, Wang YC, Mei X, Chen SY (2017). The long non-coding RNA GAS5 regulates TGF-β-induced smooth muscle cell differentiation via RNA-Smad binding element. J Biol Chem.

[44] Li Y, Maegdefessel L (2017). Non-coding RNA Contribution to Thoracic and Abdominal Aortic Aneurysm Disease Development and Progression. Front Physiol.

[45] Hill BG, Haberzettl P, Ahmed Y, Srivastava S, Bhatnagar A (2008). Unsaturated lipid peroxidation-derived aldehydes activate autophagy in vascular smooth-muscle cells. Biochem J.

[46] Tee AE, Liu B, Song R, Li J, Pasquier E, Cheung BB, Jiang C, Marshall GM, Haber M, Norris MD, Fletcher JI, Dinger ME, Liu T (2016). The long noncoding RNA MALAT1 promotes tumor-driven angiogenesis by up-regulating pro-angiogenic gene expression. Oncotarget.

[47] Luo F, Liu X, Ling M, Lu L, Shi L, Lu X, Li J, Zhang A, Liu Q (2016). The lncRNA MALAT1, acting through HIF-1α stabilization, enhances arsenite-induced glycolysis in human hepatic L-02 cells. Biochim Biophys Acta.

[48] Li L, Chen H, Gao Y, Wang YW, Zhang GQ, Pan SH, Ji L, Kong R, Wang G, Jia YH, Bai XW, Sun B (2016). Long noncoding RNA MALAT1 promotes aggressive pancreatic cancer proliferation and metastasis via the stimulation of autophagy. Mol Cancer Ther.

[49] Wang SH, Zhang WJ, Wu XC, Zhang MD, Weng MZ, Zhou D, Wang JD, Quan ZW (2016). Long non-coding RNA Malat1 promotes gallbladder cancer development by acting as a molecular sponge to regulate miR-206. Oncotarget.

[50] Lu H, He Y, Lin L, Qi Z, Ma L, Li L, Su Y (2016). Long non-coding RNA MALAT1 modulates radiosensitivity of HR-HPV+ cervical cancer via sponging miR-145. Tumour Biol.

[51] Tang Y, Jin X, Xiang Y, Chen Y, Shen CX, Zhang YC, Li YG (2015). The lncRNA MALAT1 protects the endothelium against ox-LDL-induced dysfunction via upregulating the expression of the miR-22-3p target genes CXCR2 and AKT. FEBS Lett.

[52] Xiao H, Tang K, Liu P, Chen K, Hu J, Zeng J, Xiao W, Yu G, Yao W, Zhou H, Li H, Pan Y, Li A (2015). LncRNA MALAT1 functions as a competing endogenous RNA to regulate ZEB2 expression by sponging miR-200s in clear cell kidney carcinoma. Oncotarget.

[53] Guo L, Zhao J, Qu Y, Yin R, Gao Q, Ding S, Zhang Y, Wei J, Xu G (2016). MicroRNA-20a inhibits autophagic process by targeting ATG7 and ATG16L1 and favors mycobacterial survival in macrophage cells. Front Cell Infect Microbiol.

[54] Liao XH, Wang N, Zhao DW, Zheng DL, Zheng L, Xing WJ, Ma WJ, Bao LY, Dong J, Zhang TC (2015). STAT3 Protein Regulates Vascular Smooth Muscle Cell Phenotypic Switch by Interaction with Myocardin. J Biol Chem.

